# Accidental Expulsion of a Child without Its Experiencing Any Injury from the Fall

**Published:** 1849

**Authors:** 


Artcle VII.—*Accidental Expulsion of a Child without its experiencing any injury from the fall.*

A woman of short stature, 34 years of age, strongly built, gave birth to a child in ordinary labor one year after marriage. On the 10th of July, being near the end of her second pregnancy, the period of gestation was accidently terminated. She was engaged in a violent dispute with her husband, which was nearly coming to blows: she abruptly rushed into an adjoining room, and was in the act of sitting on the bed, when suddenly strong labor came on. Before she could reach the door and call for help, the pains became so severe that she was obliged to lean for support against a chair; at the same moment the child fell suddenly to the ground, without being in the least injured. On his visit, Dr. Pickford found not even a bruise on the vertex, on which the child had fallen. The placenta was expelled as he entered, and it showed that the cord had been ruptured at the distance of two inches from the umbilicus. The child was strong and fully developed.—*Dr. Pickford, in Henle's Zeitchrift für rationelle Medizin*, vol. vii., part i., p. 25, in *Ibid.*

